# Glycocalyx-induced formation of membrane tubes

**DOI:** 10.1016/j.bpj.2025.04.006

**Published:** 2025-04-11

**Authors:** Ke Xiao, Padmini Rangamani

**Affiliations:** 1 Department of Mechanical and Aerospace Engineering, University of California, San Diego, La Jolla, California; 2 Department of Pharmacology, School of Medicine, University of California, San Diego, La Jolla, California

## Abstract

Tubular membrane structures are ubiquitous in cells and in the membranes of intracellular organelles such as the Golgi complex and the endoplasmic reticulum. Tubulation plays essential roles in numerous biological processes, including filopodia growth, trafficking, ion transport, and cellular motility. Understanding the fundamental mechanism of the formation of membrane tubes is thus an important problem in the fields of biology and biophysics. Although extensive studies have shown that tubes can be formed due to localized forces acting on the membrane or by the spontaneous curvature induced by membrane-bound proteins, little is known about how membrane tubes are induced by glycocalyx, a sugar-rich layer at the cell surface. In this work, we develop a biophysical model that combines polymer physics theory and the Canham-Helfrich membrane theory to investigate how the glycocalyx generates cylindrical tubular protrusions on the cell membrane. Our results show that the glycocalyx alone can induce the formation of tubular membrane structures. This tube formation involves a first-order shape transition without any externally applied force or other curvature-inducing mechanisms. We also find there exist critical values of glycocalyx grafting density and glycopolymer length needed to induce the formation of tubular structures. The presence of a vertical actin force, line tension, and spontaneous curvature reduce this critical grafting density and length of polymer that triggers the formation of membrane tube, which suggests that the glycocalyx makes tube formation energetically more favorable when combined with an actin force, line tension, and spontaneous curvature.

## Significance

In many cells, the existence of glycocalyx, a thick layer of polymer meshwork comprising proteins and complex sugar chains coating the outside of the cell membrane, regulates the formation of membrane tubes. Here, we propose a theoretical model that combines polymer physics theory and the Canham-Helfrich membrane theory to study the formation of cylindrical tubular protrusions induced by the glycocalyx. Analysis of this model indicates that the properties of the glycocalyx, such as length and grafting density play important roles in the formation of membrane tubes. Essentially, our work shows that the glycocalyx by itself is capable of inducing membrane tubulation, which is further facilitated when combined with actin forces, line tension, and spontaneous curvature. Thus, our theoretical model has implications for understanding how the glycocalyx may influence the formation of tubular structures on cellular membranes.

## Introduction

The generation of elongated tubular membrane geometries is ubiquitous in both plasma membrane and organelle membranes, including the endoplasmic reticulum (1,2), the Golgi apparatus (3,4), and the inner mitochondrial membrane (5,6,7). Such elongated tubular structures play important roles in numerous biological processes ranging from membrane trafficking to ion transport and cellular motility (8,9,10,11). Thus, understanding the different fundamental mechanisms of the formation of tubular membrane geometries with high curvature is an important problem in the fields of cell biology and biophysics.

In in vitro experiments, cylindrical tubes can be created using various experimental techniques including hydrodynamic flow (12), micropipette aspiration (13), and a mix of micromanipulation and optical (14) or magnetic (15) tweezers. These approaches can be attributed to a pulling force exerted on a localized point on the membrane. In vivo, additional mechanisms to induce the generation of elongated protrusions such as forces acting on the membrane by cytoskeletal assembly, filament bundles, and motor proteins (10,16,17,18,19,20,21). Beyond localized forces, there are other molecular mechanisms associated with tube formation. The interaction of cellular membranes with intrinsically curved proteins and oligomers (22) or curvature-inducing proteins (i.e., BAR [Bin/Amphiphysin/Rvs] domain proteins) without any apparent localized force mechanisms (23,24,25,26) also generate tubular structures. Recently, a wide range of studies have observed that many protrusive membrane structures such as epithelial microvilli (27,28,29,30,31,32), cilia (33), and filopodia (34,35) can be generated by mucins, a class of large, heavily glycosylated proteins that partially make up the glycocalyx. The glycocalyx is a thick layer of heavily glycosylated transmembrane macromolecules concentrated on most cell surfaces in a complex brush structure (36,37,38,39). Experimentally, to explore the mechanisms of membrane shape regulation by the glycocalyx, Shurer et al. (39) reported that bulky brush-like glycocalyx polymers are sufficient to induce a variety of curved membrane features, including spherical-shaped membranes (referred to as blebs), tubes, and unduloids, in a density-dependent manner.

Although many experimental studies indicate that the glycocalyx plays an essential role in the formation of membrane tubes, theoretical models that explicitly include the glycocalyx as a polymer layer on the membrane surface remain underdeveloped. From a theoretical point of view, the elastic continuum models based on the Canham-Helfrich theory (40,41) are frequently used to investigate the formation of membrane tubes and are well established for tubes generated through point loads (21). Meanwhile, analytical methods (42,43,44,45) and scaling theory (46) have shown that the entropic pressure exerted by polymers leads to changes in membrane shape, membrane bending moduli, and its spontaneous curvature. Using the Canham-Helfrich model and phase-field simulations (47,48), studies have demonstrated that membrane tube formation and curvature-driven pearling instabilities can be attributed to the concentration gradient of anchored polymers and the concentration of homogeneous anchored amphiphilic polymers, respectively. Furthermore, Monte Carlo simulations (49,50) have shown that high-tension polymer-anchored membranes exhibit lower curvature and that the reduction of membrane tension can lower the polymer density threshold for driving curvature. Recently, we developed a general energetic framework that couples the mechanics of the glycocalyx with the mechanics of the lipid bilayer to investigate how the glycocalyx can generate spherical vesicles and to explore whether the glycocalyx itself can sense curvature (51). Additionally, many studies have argued that using the Helfrich energy to capture tubular geometries with hemispherical caps requires a careful consideration of the curvature anisotropies (24,25). Indeed, in Zhulina et al. (52) and therefore in our work, the role of different geometries is considered explicitly. Note that the configurations of polymers vary across different membrane geometries, resulting in notable differences in the glycocalyx-associated energy. Our analysis of the literature finds that the theoretical frameworks that can describe the possible underlying mechanisms of glycocalyx-induced membrane tube formation remain lacking. To address this gap, we focus on cylinders with hemispherical caps, treating the glycocalyx as a separate entity from spontaneous curvature, with the goal of investigating how glycocalyx properties (grafting density and the length of glycocalyx) impact the properties of tubular membrane structures.

In this work, we developed a theoretical model for glycocalyx-induced membrane deformation based on polymer physics theory and the Canham-Helfrich membrane theory. Our model includes the effects of glycocalyx properties, actin force, and membrane properties on the formation of tubular membrane structures. We use the same glycocalyx polymer brush description as in (39,51), but here we do not map the glycocalyx onto the spontaneous curvature. Instead, we derive the total energy as a function of the shape parameter, glycocalyx properties, and cell membrane characteristics, assuming a family of fixed cylindrical geometries. This new total energy formulation allows for independent control over membrane-intrinsic spontaneous curvature and extraneous polymers, which is essential for precise quantitative analysis and predictions of how glycocalyx polymers remodel membrane shapes. With these new expressions for membrane-polymer composite energy, we systematically investigated the influence of the properties of the glycocalyx on the formation of membrane tubes. We find that tubular structures can be induced by glycocalyx when the glycocalyx grafting density and glycopolymer length exceed a threshold value; below this value, the formation of tubes is no longer favorable. Denser and longer glycopolymers on the membrane surface promote the formation of longer and thinner tubular structures. Cooperative effects of other energy contributions such as actin force, line tension, and spontaneous curvature serve to lower these threshold values of the glycocalyx density and length. This study presents a comprehensive energy framework, providing deeper insights into the biophysical principles that govern membrane curvature generation and has implications for understanding biological phenomena associated with the glyocalyx.

## Methods

### Description of the model

**Figure 1:**
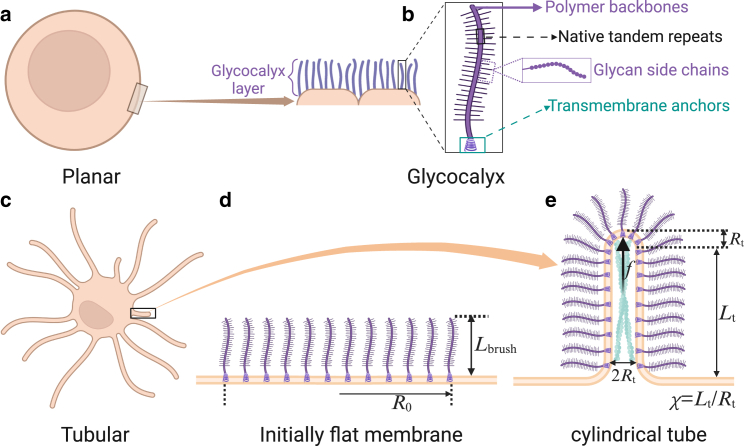
Schematic of glycocalyx-membrane system. Schematic showing (*a*) a mostly flat cell membrane surface with (*b*) a sugar-rich layer of glycocalyx coating on its outer surface. An enlarged view of the polymer structure of glycocalyx constituents such as mucins. (*c*) Sketch of tubular membrane morphology generated by the glycocalyx. (*d*) An initially flat bilayer grafted with a patch of glycocalyx of radius $R_{0}$ and brush height $L_{\text{brush}}$. (*e*) An enlarged geometrical sketch of the system: a cylindrical tube of radius $R_{t}$ and length $L_{t}$, with a hemispherical cap of radius $R_{t}$. The light blue rod inside the tube represents the cartoon schematic of a filamentous actin (F-actin) core.

We developed a biophysical model for the glycocalyx-membrane composite, where a layer of glycocalyx is coated on a membrane surface (Fig. 1, *a* and *b*). Taking mucins as an example, their polymer backbones are heavily glycosylated with O-linked sugar side chains (39,53,54,55). In our model, we consider the energy contributions of the lipid bilayer and the polymer layer independently and minimize the total energy of the system.

A recent study has shown that the entropic forces generated by the glycocalyx on the outer surface of the cell membrane can induce finger-like extensions (39). While the glycocalyx’s role in inducing spherical membrane shapes has been studied, its involvement in membrane tube formation is not well understood. In contrast to previous studies (39,48,51), the present model uniquely addresses cylindrical geometries with hemispherical membrane shapes, treating the glycocalyx separately from spontaneous curvature. To investigate the effect of glycocalyx on the generation of tubular membrane morphology (Fig. 1 *c*), we assume a membrane domain anchored by glycocalyx biopolymers with fixed surface area $A_{0}=\pi R_{0}^{2}$ is deformed into a family of cylindrical tubes with length $L_{t}$ connected with a hemispherical cap with radius $R_{t}$, where $R_{0}$ is the in-plane radius of the flat membrane, as shown in Fig. 1, *d* and *e*. In the glycocalyx-membrane system, we assume that the layer of glycopolymers is uncharged and in a brush-like structure, where the polymer brushes are extended equally in height, $L_{\text{brush}}$. We define a shape parameter, $\chi =L_{t}/R_{t}$, which characterizes the tube shape. Then, the radius of the tube can be expressed as $R_{t}=R_{0}/\sqrt{2\left(1+\chi \right)}$ under the constraint of fixed polymer grafting area $2\pi R_{t}L_{t}+2\pi R_{t}^{2}=\pi R_{0}^{2}$. When χ=0, the formation of a tubular shape is unfavorable. The membrane domain takes the shape of a family of tubes with a hemispherical cap connected when χ≠0. The energy of the polymer layer depends on the polymer properties including the grafting density, length of the polymer, and the interactions between the individual polymer chains, and also on the curvature of the underlying substrate (52). Our approach is to find the shape that corresponds to the minimal energy state for the combined energy of the membrane and the polymer for cylindrical deformations. We proceed to derive the total energy for the system, which differs from the one presented in (51). This new formulation allows us to predict the tubular morphology that glycocalyx polymers induce.

### Energetics of the system

The total free energy of the glycocalyx-membrane system $\left(F_{\text{tot}}\right)$ is modeled as the sum of four terms: the energy contribution associated with the glycocalyx $\left(F_{\text{glycocalyx}}\right)$, the elastic energy of the membrane $\left(F_{\text{membrane}}\right)$, the energy associated with a line tension $\left(F_{\text{line}\;\text{tension}}\right)$, and the work done by the vertical actin force $\left(F_{\text{force}}\right)$

(1) $$F_{\text{tot}}=F_{\text{membrane}}+F_{\text{line}\;\text{tension}}+F_{\text{force}}+F_{\text{glycocalyx}}\text{.}$$

The lipid bilayer is modeled as a thin elastic shell, which resists out-of-plane bending and we assume that the Helfrich energy is sufficient to capture the bending of the bilayer. The energy cost of bending a membrane is given by the well-known Canham-Helfrich Hamiltonian (40), which includes the bending energy of the membrane and the surface tension energy. Thus, the elastic energy of the membrane appearing in Eq. 1 can be written as

(2) $$F_{\text{membrane}}=\int_{A_{\text{mem}}}\frac{\kappa }{2}{\left(2H-c_{0}\right)}^{2}\;dA+\sigma \Delta A\text{,}$$

where κ is the membrane bending rigidity, $H=\left(c_{1}+c_{2}\right)/2$ is the mean curvature in which $c_{1}$ and $c_{2}$ are the two principal curvatures, $c_{0}$ is the spontaneous curvature, σ is the membrane tension, and ΔA is the change in the in-plane area due to the membrane shape deformation. Here, $c_{0}$ is the spontaneous curvature which is restricted to asymmetries in the bilayer. In the following, we analyze the effects of the glycocalyx on curvature generation independently of spontaneous curvature, taking into account the energy contribution associated with the glycocalyx.

Recently, Lu et al. (53) demonstrated that MUC1 can sense membrane curvature and redistribute on the plasma membrane, owing to the physical attachment of the glycocalyx to the cell membrane. In addition, Gollapudi et al. (54) showed that transmembrane fusion proteins with the ectodomain of MUC1 can diffuse on the plasma membrane and be recruited to endocytic structures. Based on these observations, we assume that these transmembrane proteins diffuse laterally along the membrane and can aggregate into domains. To take into account the multiple phases on the membrane, for example, the glycocalyx rich domain and the surrounding bare membrane, we also considered the effect of the line tension at the glycopolymer’s anchored domain boundary or interface ∂l. Therefore, the second term on the right-hand side of Eq. 1 is the interfacial energy or the energy associated with the line tension and is given by

(3) $$F_{\text{line}\;\text{tension}}=\lambda \oint_{\partial l}dl\text{,}$$

where λ denotes the strength of line tension along the domain boundary, and the integral is over the periphery line dl of the domain. The integral is over the periphery line dl of the membrane patch on which the glycocalyx is anchored. We assume that the value of line tension is a constant. Intuitively, this line tension describes the energetic cost of maintaining a high-density and a low-density region on the membrane.

The third term on the right-hand side of Eq. 1 describes the work done by the vertical actin force. The polymerization of actin filaments can generate pushing force for protrusion in living cells (18,56,57). Notably, Shurer et al. (39) showed that the polymerization and depolymerization of cytoskeletal filamentous actin (F-actin) core (see Fig. 1 *e*) also plays a key role in mediating membrane shape. Because the precise architecture formed by the F-actin core inside the cell and the resulting forces are not yet well established, we assume that the F-actin cores form bundles (rods) that apply vertical forces on the membrane. Note that we assume that the vertical force is a point force (21,58,59,60) in the z direction in our model. Based on such an assumption, we model the force generated by the F-actin core, f, as

(4) $$F_{\text{force}}=-fZ\text{,}$$

where Z is the height of the deformed membrane patch.

We restrict the membrane geometry to a cylindrical tube connected with a spherical cap. The two principal curvatures for the cylindrical tube are given by $c_{1}=0$ and $c_{2}=1/R_{t}$, and for the spherical cap are given by $c_{1}=c_{2}=1/R_{t}$. As a result, the excess area is calculated as $\Delta A=\left(2\chi +1\right)\pi R_{t}^{2}$, the length of the domain boundary can be written as $\oint_{\partial l}dl=2\pi R_{t}$, and the height of the deformed membrane patch is given by $Z=R_{t}+L_{t}$. Therefore, the sum of the elastic energy of the membrane, the line tension energy, and the work done by the vertical actin force becomes

(5) $$\frac{F_{\text{membrane}}+F_{\text{line}\;\text{tension}}+F_{\text{force}}}{\pi \kappa }=\frac{F_{\text{bending}}+F_{\text{membrane}\;\text{tension}}+F_{\text{line}\;\text{tension}}+F_{\text{force}}}{\pi \kappa }=\underset{\text{bending}}{\underset{︸}{\chi R_{t}^{2}{\left(\frac{1}{R_{t}}-c_{0}\right)}^{2}+R_{t}^{2}{\left(\frac{2}{R_{t}}-c_{0}\right)}^{2}}}+\underset{\text{membrane}\;\text{tension}}{\underset{︸}{\frac{\sigma }{\kappa }\left(2\chi +1\right)R_{t}^{2}}}+\underset{\text{line}\;\text{tension}}{\underset{︸}{\frac{2\lambda }{\kappa }R_{t}}}\underset{\text{mechanical}\;\text{work}}{\underset{︸}{-\frac{fR_{t}}{\pi \kappa }\left(1+\chi \right)}}=\left[\left(\chi +1\right)c_{0}^{2}+\frac{\sigma }{\kappa }\left(2\chi +1\right)\right]R_{t}^{2}+2R_{t}\left(\frac{\lambda }{\kappa }-\chi c_{0}-2c_{0}\right)+\chi +4-\frac{fR_{t}}{\pi \kappa }\left(1+\chi \right)\text{,}$$

where dividing by πκ normalizes the energy.

The final term in Eq. 1 is the energy contribution associated with the glycocalyx, which includes the elastic stretching of the polymer chains and the interactions between monomers in the polymer brush. Based on (52), the sum of the energy density of these two terms for a single polymer is given by

(6) $$f_{\text{glycocalyx}}^{\text{elastic}}+f_{\text{glycocalyx}}^{\text{EV}}=\frac{1}{\beta }\left[\frac{3}{2a^{2}c_{p}\left(r\right)s^{2}\left(r\right)}+vc_{p}^{2}\left(r\right)+wc_{p}^{3}\left(r\right)+\ldots \right]\text{,}$$

where $\beta =1/\left(k_{B}T\right)$ with $k_{B}T$ being the unit thermal energy, a is the monomer size, $c_{p}\left(r\right)$ is the local monomer density profile along the thickness, r is the radial distance defined from the center of the spherical or cylindrical surface, s(r) is the area per chain at distance from the polymer grafting surface, and v and w are the second and third virial coefficients, respectively. The dependence of $c_{p}\left(r\right)$ and s(r) on the surface shape means that the energy contributions associated with the glycocalyx differ between cylinders and spheres. Our previous work (51) addressed spherical geometries, whereas here we examine cylinders with hemispherical caps. Integrating this energy density from $R_{t}$ to $R_{t}+L_{\text{brush}}$ and then multiplying the total number of grafted polymers for the cylindrical tube and the hemispherical cap, respectively, leads to the total energy contribution from glycocalyx polymers

(7) $$\begin{array}{cc} \frac{F_{\text{glycocalyx}}}{\pi \kappa } & =\frac{F_{\text{glycocalyx}}^{\text{cap}}+F_{\text{glycocalyx}}^{\text{tube}}}{\pi \kappa }\\ & =N_{p}^{\text{cap}}\frac{9R_{t}}{2\pi \beta \kappa }{\left(\frac{3\sqrt{v}}{\xi a^{2}}\right)}^{\frac{2}{3}}\left[{\left(1+\frac{5N}{3R_{t}}\cdot {\left(\frac{va^{2}}{3\xi^{2}}\right)}^{\frac{1}{3}}\right)}^{\frac{1}{5}}-1\right]\\ & +N_{p}^{\text{tube}}\frac{9R_{t}}{4\pi \beta \kappa }{\left(\frac{3\sqrt{v}}{\xi a^{2}}\right)}^{\frac{2}{3}}\left[{\left(1+\frac{4N}{3R_{t}}\cdot {\left(\frac{va^{2}}{3\xi^{2}}\right)}^{\frac{1}{3}}\right)}^{\frac{1}{2}}-1\right]\text{,} \end{array}$$

where $N_{p}^{\text{cap}}$ and $N_{p}^{\text{tube}}$ are the number of polymer chains that grafted on the spherical cap and the cylindrical tube, ξ is the grafting distance, which is related to the grafting density ρ via $\rho =1/\xi^{2}$, and N is the number of monomers in a polymer chain. Here, the relation $\chi =L_{t}/R_{t}$ is used, and the detailed derivation of this individual energy component (Eq. 7) is provided in the supporting material. Note that only the pairwise monomer-monomer interactions with second virial coefficient v is considered, and that the ternary interactions with third virial coefficient w are neglected.

Finally, combining Eqs. 5 and 7, and using $N_{p}^{\text{cap}}=2\pi R_{t}^{2}/\xi^{2}$ and $N_{p}^{\text{tube}}=2\pi R_{t}L_{t}/\xi^{2}$, the total free energy of the glycocalyx-membrane system with a cylindrical tube of radius $R_{t}$ and length $L_{t}$, and with a hemispherical cap of radius $R_{t}$ can be obtained as

(8) $$\begin{array}{cc} \frac{F_{\text{tot}}}{\pi \kappa } & =\frac{F_{\text{membrane}}+F_{\text{line}\;\text{tension}}+F_{\text{force}}+F_{\text{glycocalyx}}}{\pi \kappa }\\ & =\left[\left(\chi +1\right)c_{0}^{2}+\frac{\sigma }{\kappa }\left(2\chi +1\right)\right]R_{t}^{2}+2R_{t}\left(\frac{\lambda }{\kappa }-\chi c_{0}-2c_{0}\right)\\ & +\chi +4-\frac{fR_{t}}{\pi \kappa }\left(1+\chi \right)\\ & +\frac{9R_{t}^{3}}{2\beta \kappa \xi^{2}}{\left(\frac{3\sqrt{v}}{\xi a^{2}}\right)}^{\frac{2}{3}}\{2\left[{\left(1+\frac{5N}{3R_{t}}\cdot {\left(\frac{va^{2}}{3\xi^{2}}\right)}^{\frac{1}{3}}\right)}^{\frac{1}{5}}-1\right]\\ & \;+\chi \left[{\left(1+\frac{4N}{3R_{t}}\cdot {\left(\frac{va^{2}}{3\xi^{2}}\right)}^{\frac{1}{3}}\right)}^{\frac{1}{2}}-1\right]\}\text{.} \end{array}$$

Hereafter, in our model, we use the number of monomers, N, to capture the length of the polymer due to the thickness of the polymer brush is related to the total number of monomers N (see Eqs. S9 and S12). We assume that our system is at equilibrium, implying that the system selects the membrane shape that minimizes the total free energy of Eq. 8.

### Numerical implementation

Here, we are focused on the equilibrium state of the system, so our objective is to determine the global minimum energy state by numerically calculating the corresponding total free energy of the system. To numerically calculate the total free energy, the excluded volume parameter is set as $v=a^{3}$ as suggested by (52) for neutral brushes. We have combined these contributions from the glycocalyx polymers and the cell membrane to construct the total free energy of the membrane patch with fixed area $A_{0}$. Minimization of the total energy as a function of shape parameter χ yields the optimal $\chi_{\min}$ that determines the morphology of the membrane patch. Biologically relevant values for the parameters that have been used in the mathematical model are summarized in Table 1. The code is available on https://github.com/RangamaniLabUCSD/Glycocalyx_membrane-tubes.

**Table 1:** Values of the different parameters used in the model

Parameter	Meaning	Estimated Range	References	Default Value
κ	bending rigidity	$10-400\;k_{B}T$	(61,62,63,64)	$10\;k_{B}T$
σ	membrane tension	$10^{-7}-10^{-3}\;N/m$	(62,65,66)	$0.012\;k_{B}T/{\text{nm}}^{2}$
λ	line tension	0−100pN	(62,67,68)	$0.5\;k_{B}T/\text{nm}$
$R_{0}$	patch radius	20−100nm	(50,62)	100nm
ξ	grafting distance	10−100nm	(39,69)	15nm
a	monomer size in the glycocalyx	10−20nm	(36,39,50,70,71,72)	10nm
N	number of monomers in a brush	10−50	(36,39,50,72)	20
$c_{0}$	spontaneous curvature	$0-0.1\;{\text{nm}}^{-1}$	(39)	$0.02\;{\text{nm}}^{-1}$
f	vertical axial actin force	$0-5\;k_{B}T/\text{nm}$	(58,73,74,75,76,77)	$1\;k_{B}T/\text{nm}$

## Results

To unveil the effects of glycocalyx properties, membrane properties, and actin force on the formation of membrane tubular, we numerically calculate the total energy for different shape parameters χ by varying each of these parameters individually. The global energy minimum corresponds to the optimal $\chi_{\min}$ that gives the equilibrium geometry of the glycocalyx-covered membrane patch. We used the default parameter values listed in Table 1, unless otherwise indicated.

### Effect of glycocalyx on membrane tubular formation

**Figure 2:**
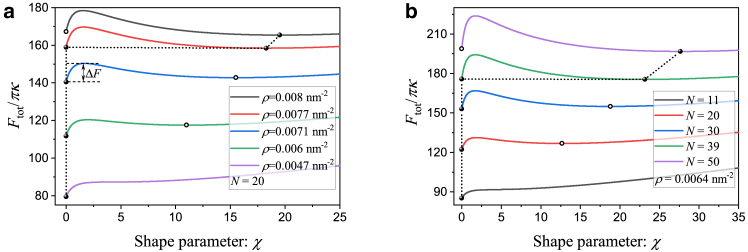
Total energy profile as a function of shape parameter χ for different values of (*a*) glycopolymer density ρ and (*b*) glycopolymer length N, where the bending modulus, membrane tension, line tension, spontaneous curvature, and vertical force are fixed at $\kappa =10\;k_{B}T$, $\sigma =0.012\;k_{B}T/{\text{nm}}^{2}$, $\lambda =0\;k_{B}T/\text{nm}$, $c_{0}=0\;{\text{nm}}^{-1}$, and $f=0\;k_{B}T/\text{nm}$, respectively.

To investigate the effects of the glycocalyx properties including grafting density and length, we first perform the analysis of total energy for different values of grafting density ρ and length N, as shown in Fig. 2.

In Fig. 2 *a*, we plot the total energy as a function of the shape parameter χ for different grafting densities. Here, the corresponding contributions from different energy components are plotted in Fig. S2. Note that the total energy is a nonlinear function of the shape parameter. The energy curve shows a monotonic behavior when the glycopolymer grafting density is smaller than a critical value (see the *purple curve* $\rho =0.0047\;{\text{nm}}^{-2}$), where there is only one local minimum (global minimum) at χ=0 (*solid circle* in the *purple curve*), corresponding to zero tube length. This indicates that, when the glycocalyx is sparsely distributed on the membrane, tube formation is not energetically favorable. As grafting density increases, the energy profile corresponds to two local minima: one stable nontubular state (χ=0), and another metastable tubular state (χ≠0). This suggests that the initial spherical cap shape is still more favorable compared with the tubular structure (see the *green* and *blue curves*). If the grafting density is larger than a critical value $\left(\rho =0.0077\;{\text{nm}}^{-2}\right)$, the total energy of the nontubular state becomes equal to that of the tubular state (see the *red curve*), indicating that the two states coexist. Further increase of grafting density leads to the transition from a nontubular state to a tubular state (see the *black curve*), which implies that the initial spherical cap becomes unstable against the formation of a tube. Note that the initial spherical cap, to grow up to its preferred length (*solid point* on the *black curve*), needs to overcome an energy barrier ΔF. This observation is consistent with the conclusion of previous studies (48). Campelo and Hernández-Machado (48) argue that polymer-induced tubulation is caused by the nonhomogeneous concentration of amphiphilic molecules anchored on the membrane. Both the present work and (48) assume a cylindrical tube with a hemispherical cap and demonstrate that tubular structures can be induced by polymers anchored on the membrane within the framework of the Canham-Helfrich model for membrane energy. However, a direct comparison between these two works reveals several key differences. Specifically 1) Campelo and Hernández-Machado (48) assumed a linear coupling between spontaneous curvature and local polymer concentration, whereas we assume that polymers are uniformly grafted on the plasma membrane without mapping the glycocalyx onto spontaneous curvature. 2) Our model incorporates polymer length and grafting density as parameters to study the impact of glycocalyx on membrane shape remodeling. 3) Instead of relying on spontaneous curvature assumptions, we directly derive the glycocalyx’s energy contributions and also consider the energies associated with line tension and the vertical actin force.

In addition to grafting density, length of the polymers is another important feature of the glycocalyx layer on the cell membrane surface. To probe the influence of glycopolymer length on the formation of membrane tube, the analysis of total energies for various numbers of monomer N is presented in Fig. 2 *b*. Upon increasing N, the energy profiles in Fig. 2 *b* show a similar trend as presented in Fig. 2 *a*, indicating that longer glycopolymer length can trigger a state transition from nontubular to tubular (i.e., the *purple curve*, N=50). Thus, the glycopolymer length also has a big impact on the formation of membrane tubes. The finding of the tube shape induced by the glycocalyx is in line with those observed experimentally by Shurer et al. (39). In relation to Shurer et al. (39), our work extends their theory by deriving glycocalyx energy contributions under specific membrane shape assumptions. While Shurer et al. (39) introduces the conceptual framework, it does not provide explicit expressions for the total energy. In contrast, our theoretical model, while utilizing the same glycocalyx polymer brush model, derives the total energy as a function of the shape parameter, glycocalyx properties, and cell membrane characteristics under the assumption of a family of fixed-membrane geometries. This formulation facilitates precise quantitative analysis and predictions of glycocalyx-induced membrane shape remodeling.

### Discontinuous transition from nontubular state to tubular state

**Figure 3:**
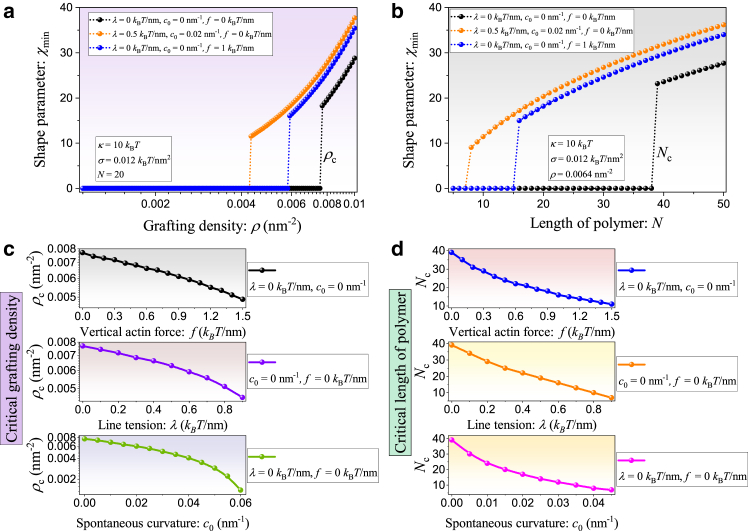
The dependence of minimum shape parameter $\chi_{\min}$ on glycopolymer density ρ with N=20 and (*b*) glycopolymer length N with $\rho =0.0064\;{\text{nm}}^{-2}$, where the bending modulus and membrane tension are fixed at $\kappa =10\;k_{B}T$ and $\sigma =0.012\;k_{B}T/{\text{nm}}^{2}$, respectively. The dependence of (*c*) critical grafting density, $\rho_{c}$, and (*d*) critical length of polymer, $N_{c}$, on actin force (*top row*), line tension (*middle row*), and spontaneous curvature (*bottom row*).

We next focused on the stable state corresponding to the shape parameter $\chi_{\min}$. Fig. 3 shows the dependence of $\chi_{\min}$ on grafting density and glycopolymer length.

In the absence of line tension λ, spontaneous curvature $c_{0}$, and actin force f, the black dotted curves in Fig. 3, *a* and *b* illustrate that the optimal shape parameter shows a sharp jump at the critical values, $\rho_{c}$ or $N_{c}$, a characteristic of discontinuous transition. Such a transition indicates that the glycocalyx alone is able to induce the membrane to form a stable tubular morphology. Furthermore, to investigate the influences of line tension λ, spontaneous curvature $c_{0}$, and actin force f on the dependence behaviors of $\chi_{\min}$ on glycopolymer density ρ and length N, the orange and blue curves in Fig. 3, *a* and *b* show that the discontinuous transition is still maintained but with a shift of the threshold $\rho_{c}$ or $N_{c}$, which triggers the discontinuous transition from nontubular state to tubular state. Moreover, we found that the presence of line tension λ, spontaneous curvature $c_{0}$, and actin force f results in the decreases of the critical value $\rho_{c}$ or $N_{c}$, as shown in Fig. 3, *c* and *d*. Also, we can generate longer and thinner membrane tubes (larger $\chi_{\min}=L_{t}/R_{t}$) in the presence of line tension λ, spontaneous curvature $c_{0}$, and actin force f. Consequently, we can infer that the presence of line tension, spontaneous curvature, and actin force plays a role in relaxing the critical conditions for the formation of membrane tubes. Theoretical predictions in Shurer et al. (39) also reveal a discontinuous behavior, as Shurer et al. showed that the equilibrium point force required to maintain an extended membrane tubule decreases with increasing mucin concentration. Notably, this force undergoes a discontinuous decrease as the mucin concentration approaches the mushroom-brush transition boundary.

### Phase diagrams for glycocalyx-induced tubular formation

**Figure 4:**
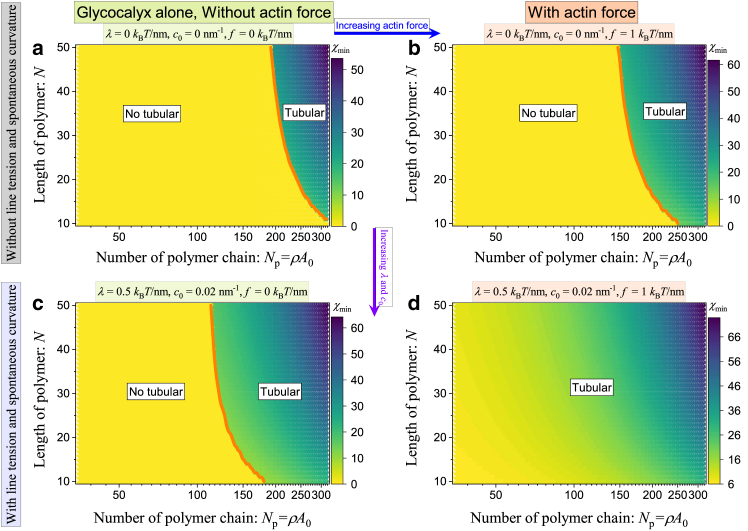
Phase diagrams present the optimal shape parameter $\chi_{\min}$ as a function of number of polymer chain, $N_{p}$, and length of polymer, N, for the situations of (*a*) without line tension $\lambda =0\;k_{B}T/\text{nm}$, spontaneous curvature $c_{0}=0\;{\text{nm}}^{-1}$, and vertical actin force $f=0\;k_{B}T/\text{nm}$; (*b*) with actin force $f=1\;k_{B}T/\text{nm}$, and without line tension $\lambda =0\;k_{B}T/\text{nm}$ and spontaneous curvature $c_{0}=0\;{\text{nm}}^{-1}$; (*c*) with line tension $\lambda =0.5\;k_{B}T/\text{nm}$ and spontaneous curvature $c_{0}=0.02\;{\text{nm}}^{-1}$, and without vertical actin force $f=0\;k_{B}T/\text{nm}$; (*d*) with line tension $\lambda =0.5\;k_{B}T/\text{nm}$, spontaneous curvature $c_{0}=0.02\;{\text{nm}}^{-1}$, and vertical actin force $f=1\;k_{B}T/\text{nm}$.

To systematically study the interrelated effects of glycopolymer density and length on the membrane shape, we summarize the observed stable states in phase diagrams presented in Fig. 4 for four different cases: 1) in the absence of line tension λ, spontaneous curvature $c_{0}$, and actin force f (see Fig. 4 *a*), 2) in the presence of actin force f and in the absence of line tension λ and spontaneous curvature $c_{0}$ (see Fig. 4 *b*), 3) in the absence of actin force f and in the presence of line tension λ and spontaneous curvature $c_{0}$ (see Fig. 4 *c*), and 4) in the presence of line tension λ, spontaneous curvature $c_{0}$, and actin force f (see Fig. 4 *d*).

Fig. 4 shows that two regimes corresponding to nontubular and tubular states are identified, in which the color indicates the value of the optimal shape parameter $\chi_{\min}$. The discontinuous transition from a nontubular state to a tubular state can be triggered by tuning the grafting density and length of the glycocalyx. Fig. 4 *a* revealed that, even without the line tension, spontaneous curvature, and actin force, the glycocalyx alone can regulate the formation of tubular structures under the condition of crossing the threshold of the grafting density and polymer length. When comparing Fig. 4 *a* with Fig. 4 *b*, we observe that the inclusion of vertical actin force leads to a broader tubular regime and shifts the boundary curve to the left (see the *orange curve*). This validates the prediction presented in the top row in Fig. 3, *c* and *d*, which is that lower $\rho_{c}$ or $N_{c}$ is sufficient to induce the formation of membrane tube when other factors are present. Furthermore, the comparison of the tubular region between Fig. 4 *a* and Fig. 4 *c* shows that the tubular regime becomes wider in the presence of line tension and spontaneous curvature. This confirms the predictions illustrated in the middle and bottom rows of Fig. 3, *c* and *d*, which show that the threshold for inducing the formation of tubules decreases as the line tension and spontaneous curvature increase. In the end, a direct comparison of the tubular region in Fig. 4 *d* with that of Fig. 4 *a* (or Fig. 4, *b* and *c*) implies that the line tension, spontaneous curvature, and actin force facilitate the formation of tubules in conditions that would not be favored otherwise. Fig 4 *d* also shows that the unfavorable conditions (no tubular regime) vanish in the selected parameter space when line tension, spontaneous curvature, and actin force are taken into account. We expect that a regime unfavorable to tube formation will emerge when the polymer grafting density and polymer length are sufficiently low.

In general, we find the existence of two stable states in which the system can reside: in the nontubular state, the initial spherical cap cannot grow into a tubular shape; in the tubular state, a stable tube can be formed after crossing an energy barrier in the presence of glycocalyx. In our theoretical model, the total free energy Eq. 8 includes the contribution of the elastic energy of the membrane, which indicates that membrane shape remodeling is also impacted by membrane properties such as bending rigidity and membrane tension. This prompts us to explore the effects of the membrane properties on tube formation. The corresponding two-dimensional phase diagram on the (κ−σ) plane to characterize the interrelated effects of membrane bending rigidity and membrane tension on the membrane shape is presented in Fig. S3 *a*. Also, to intuitively observe the influence of line tension, spontaneous curvature, and vertical actin force on tube formation, two more phase diagrams on the $\left(\lambda -c_{0}\right)$ and $\left(f-c_{0}\right)$ planes are constructed, as shown in Fig. S3, *b* and *c*. Our results demonstrate that tubule formation is more easily achieved under conditions of lower membrane bending rigidity and tension. Moreover, the presence of line tension, spontaneous curvature, and actin force not only relaxes the critical conditions necessary for the formation of tubular structures but also promotes the generation of longer and thinner tubes.

We acknowledge that there are several similarities in the theoretical framework between this work, our previous work (51), and the theory proposed by Shurer et al. (39). However, we present several new findings: 1) explicit expressions for the energy contributions for a lipid bilayer with a layer of glycocalyx grafted on a cylindrical geometry are derived, 2) the work done by the actin force due to the polymerization of actin filaments is incorporated into the current model, 3) the dependence of critical grafting density and critical polymer length on various parameters is investigated independent of the spontaneous curvature, and 4) An energy barrier is identified that need to be overcome to form tubules from an initially hemispherical cap. Based on the theoretical model, we predict that tubular structures can also emerge as a possible morphology generated by the glycocalyx, with critical conditions required to trigger the onset of tubulation.

## Discussion

By combining polymer physics theory and Helfrich membrane theory, here we propose a theoretical model to elucidate the physical origin behind the glycocalyx-regulated membrane tube formation. Consequently, we argue that glycocalyx plays a significant role in generating membrane tubes. To do so, critical values of grafting density and polymer length need to be reached to induce a tubular membrane by the glycocalyx. The presence of line tension, spontaneous curvature, and actin force can then further promote the formation of membrane tubes. The physics behind the transition between the nontubular state and tubular state stems from the competition among the glycocalyx energy, the elastic energy (consisting of bending energy and tension energy), and the work done by the vertical actin force. Membrane bending is facilitated by forces due to the glycocalyx and actin, line tension, and spontaneous curvature, whereas membrane elasticity counteracts its deformation.

In summary, based on the developed theoretical framework, we study the membrane tube formation regulated by glycocalyx and find that the presence of glycocalyx is able to trigger a discontinuous nontubular to tubular transition. Such a transition provides a deeper insight into the membrane tube formation behaviors induced by glycocalyx. We identify that the tube length in the tubular regime can be increased upon regulating many parameters, such as increasing glycocalyx grafting density and length, line tension and spontaneous curvature, and actin force, reducing membrane bending rigidity and tension. Therefore, aside from the polymerization of actin filament bundles against a membrane, the interaction of cellular membranes with proteins that induce curvature, and a pulling force exerted on a membrane, the presence of the glycocalyx, a sugar-rich layer at the cell surface, is another different necessary biophysical condition for the formation of tubular membrane protrusions, particularly on cellular membranes. Taken together, the entropic bending force generated by the glycocalyx plays a crucial role in the formation of curved membrane structures. Given the high grafting density of the polymer brushes in the glycocalyx layer, collisions between adjacent polymer chains stretch the chains perpendicularly to the membrane surface (39,78). This stretching leads to a decrease of the chain entropy and a related increase of the chain free energy (39,78). To reduce the entropy cost, the membrane tends to bend in the direction of the polymers for the sake of creating more space for the polymer chains and alleviating the polymer chain stretching. As a result, a repulsive interaction between the chains (or an effective entropic bending force) is exerted on the membrane, leading to membrane bending due to the accumulated entropic free energy caused by the stretching of the membrane-grafted polymer brushes.

The glycocalyx is a sugar-rich layer that serves as a physical barrier between the cell and its surrounding environment, providing protective functions for the cell membrane, such as shielding the cell from invasion by disease pathologies or foreign objects. It has been shown that the glycocalyx plays a vital role in respiratory viral infections and overall health (30,53). In many cancers, disease aggression and poor patient prognosis are associated with an abundance of mucins on the cell surface, where the enhanced expression of mucins correlates with unique membrane features (36,39,79). Experimentally, there is evidence that MUC1 and other large glycosylated proteins frequently accumulate at high densities on protrusive membrane structures, such as filopodia (34,35), microvilli (27,28,29,30,31,32), and cilia (33). On one hand, changes in the properties of the glycocalyx can generate membrane curvature, which is linked to various cell surface structures as well as the release of extracellular vesicles. On the other hand, it is possible for cells to regulate glycocalyx properties to control tubulation because cells can dynamically change the composition and architecture of the glycocalyx to modulate their behavior (80). For example, many processes, including the regulation of glycan-related transcripts, modulation of secretory system flux, and rewiring of cellular metabolism, are involved in generating the necessary building blocks for glycan biosynthesis (80). Given this, cellular states and behaviors are closely coupled with the properties of the glycocalyx. Additionally, the intact structure of the glycocalyx is closely related to inflammation. The blood-brain barrier system primarily consists of pericytes, endothelial cells, the glycocalyx, the basement membrane, and astrocyte cells (81). The glycocalyx plays a crucial role in many essential physiological functions, including vascular permeability, inflammation, blood coagulation, and the synthesis of nitric oxide (81). As a result, damage to the glycocalyx may lead to various pathologies. For example, if the glycocalyx is degraded, leukocytes and platelets are more likely to bind to receptors on endothelial cells, leading to inflammation, blood clotting, cerebral microcirculation ischemia, and damage to nervous tissue (81,82,83). In a mouse model of inflammation, it has been observed that destruction of the vascular endothelial glycocalyx structure induces a series of inflammatory and pathological changes (84,85,86,87,88,89).

So far, numerous theoretical models have been developed to gain a better understanding of the formation of tubular structures generated by various mechanisms (18,21,24,25,26,48,90). However, our model differs from other models in the following aspects. In our model, tubulation can be generated without a directed force acting on the membrane when it is coated with a layer of glycocalyx, in contrast to previously reported cases (18,21,90). In addition to the point-like force applied to the membrane, several studies have shown that tubular shapes can also be induced by anisotropic curvature-inducing proteins coating on the membrane (24,25,26). In comparison with that protein scaffolding mechanism, here we show a novel mechanism of tube extraction that bulky brush-like glycocalyx polymers are sufficient to induce the formation of a cylindrical tube in the absence of any curvature-inducing proteins. Of note is the study by Campelo and Hernández-Machado (48), which shows that the formation of a cylindrical tubule is ascribed to the presentation of a polymer concentration gradient. In addition, the work from Shurer et al. (39) demonstrated that the spontaneous curvature induced by the glycocalyx can cooperate with external point forces to generate membrane tubes. In this work, we show that the elongation of a hemispherical cap membrane into a cylindrical tube may be energetically favorable when critical values of glycocalyx grafting density and glycopolymer length are reached. Additionally, our developed model allows us to investigate the effects of glycocalyx properties (grafting density and length of glycocalyx) on the formation of tubular membrane structures.

Based on our theoretical results, we make the following experimentally relevant predictions. The formation of a cylindrical tubule induced by the glycocalyx depends on its properties and requires to reach the threshold grafting density and glycopolymer length. Two distinct stable states are found, depending on the characteristics of the glycocalyx: a tubular state for high grafting density and long polymer length, and a nontubular state for low grafting density and short polymer length. Experimentally, this prediction can be tested by conducting experiments to observe the structures induced on Muc1-42TR-expressing cells (39) by varying the mucin density. The impact of polymer length on tube formation can also be verified, as it is experimentally feasible to tune the number of tandem repeats of Muc1 (39,53,54). For a given glycocalyx grafting density and thickness, the assistance of line tension, spontaneous curvature, and actin force is conducive to tube formation. This prediction could imply that actin polymerization (16,17,18) and the action of motor proteins (19,20) on the membrane will make the plasma membrane more favorable to tubulation in living cells. The predictions of our model have implications for understanding the formation of tubular structures generated by the glycocalyx in cells. For example, many unique membrane features in tumor cells are associated with the expression of mucins and hyaluronan on their surface (35,91,92) and the high incidence of tubular protrusions at the micro- and nanoscale from the cell membrane in various cancer phenotypes (93). We expect that the findings from our work will contribute to a better understanding of the role of individual glycocalyx properties in regulating membrane shapes and provide deeper insights into how membrane tubes form under various biophysical conditions.

We note that our present work has some limitations and simplifications, such as prescribing the shape of the membrane as a simplified geometrical scheme, treating the glycocalyx as an uncharged polymer brush network, and neglecting the spatial heterogeneity of the distribution of the glycocalyx polymers on the membrane. Since mucins are typically negatively charged (39,78), we speculate that the electrostatic repulsive forces generated by the negative charges in the glycocalyx simply serve to enhance the steric effects. To investigate the entropic interactions alone, we excluded the effects of electrostatic interactions by assuming the polymers to be uncharged. Beyond filament polymerization and depolymerization, the attachment of the membrane to the actin cortex may also suppress glycocalyx-mediated membrane morphologies within the cell (94,95,96). Future modeling and experimental studies could consider incorporating the diffusion of glycocalyx polymers, changes in the bulk physical properties of the glycocalyx, as well as the effects of membrane-actin cortex attachment and charged polymers to better capture the biological complexity of cell membranes.

## Acknowledgments

This work was supported by 10.13039/100000002NIH R01GM132106, 10.13039/100000152NSF MCB 2327243, and 10.13039/100000006Office of Naval Research N00014-20-1-2469 to P.R. We thank Dr. Emmett Francis for proofreading the manuscript and giving valuable feedback.

## Author contributions

K.X. and P.R. conceptualized the study. K.X. and P.R. performed the calculations and analyzed the results. All authors contributed to writing the manuscript. Both authors reviewed and edited the manuscript.

## Declaration of interests

P.R. is a consultant for Simula Research Laboratories in Oslo, Norway, and receives income. The terms of this arrangement have been reviewed and approved by the University of California, San Diego, in accordance with its conflict of interest policies.

## Supporting material

Document S1. Figures S1–S3

Document S2. Article plus supporting material
