# Supplementary material for: Glycocalyx-induced formation of membrane tubes
Source: Biophys J. 2025 Apr 11;124(10):1631–42. doi: 10.1016/j.bpj.2025.04.006 (PMC12242410; doi:10.1016/j.bpj.2025.04.006)
Supplement: Document S1. Figures S1–S3 [file mmc1.pdf]

Biophysical Journal, Volume *124*

Supplemental information

Glycocalyx-induced formation of membrane tubes

Ke Xiao and Padmini Rangamani

Supplementary Materials for "Glycocalyx-induced formation of membrane tubes"

Ke Xiao[1] and Padmini Rangamani[1,2]*

[1]Department of Mechanical and Aerospace Engineering, University of California San Diego, La Jolla CA 92093, USA.

[2]Department of Pharmacology, School of Medicine, University of California San Diego, La Jolla CA 92093, USA.

*To whom correspondence must be addressed: prangamani@health.ucsd.edu

1 Derivation of the energy contribution associated with the glycocalyx

The crowding of large glycosylated proteins appears to regulate the shape of the underlying bilayer plasma membrane (*1*). According to polymer physics, the glycocalyx polymers on cell membrane surfaces exhibit two regimes depending on their grafting density, which are the mushroom-like regime and the brush-like regime. In the case of high-density glycocalyx polymers, Shurer et al. (*1*) have reported that the mucins are in the brush-like structure which is able to regulate membrane morphology. To model the influence of the glycocalyx on cell membrane morphology, in our theoretical model, we focus on densely grafted regions of the membrane and therefore model the glycocalyx in the brush regime.

Here, we consider a layer of glycocalyx (brush-like structure) grafted on a membrane with a cylindrical tube and a hemispherical cap geometry, where the tube and cap radii are R_t, the tube length is L_t, and the thickness of the brush-like structure is L_{brush}, as shown in Fig. S1. From the

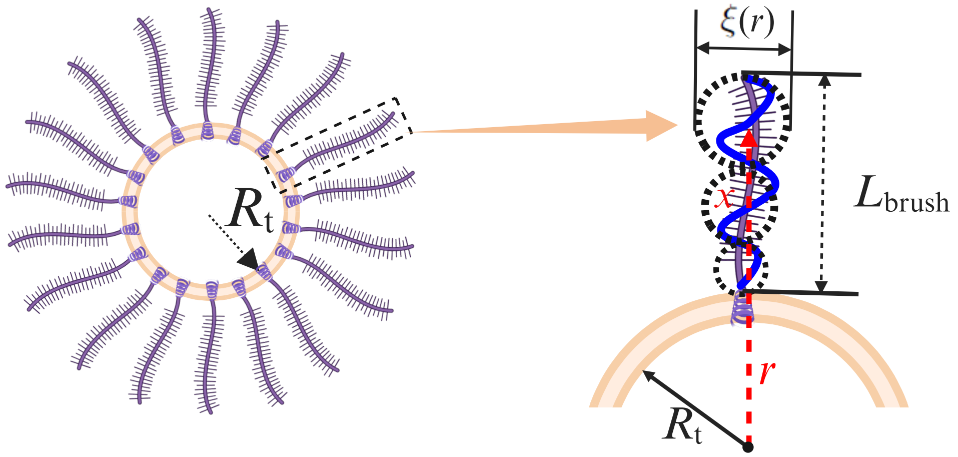

Figure S1: (i) Schematic of the cross-section of a cylindrical tube or a sphere membrane grafted with glycocalyx. (ii) An enlarged schematic illustration of fragments of a polymer chain anchored on a cylinder or sphere with the radius of curvature R_t. At a given position r, the coarse grained blob (dashed black circle) size in a polymer brush is $\xi(r)$, and the thickness of the brush is L_{brush}.

viewpoint of coarse-graining, the polymer brush is envisioned as an array of blobs. The size of each blob, ξ, at a given position r, equals the square root of the local area per chain $s(r)$, where $r = x + R_t$ is the radial distance which is defined from the center of the spherical surface or the cylindrical tube surface, and in which x is the distance from the membrane surface. Thus, the blob

size $\xi = \sqrt{s(r)}$ grows as a function of r, and the grafting density of glycocalyx polymer brush on the membrane surface can be obtained as $\rho = 1/\xi^2$. Assuming that the layer of glycopolymers is extended non-uniformly but equally in the height, L_{brush}, then the area per chain at distance x from the membrane surface is given by (*2*)

$$s(r) = s(x + R_t) = s(R_t)\left(\frac{r}{R_t}\right)^{i-1}, \quad (i = 1, 2, 3). \tag{S1}$$

The index $i = 1,\ 2,\ 3$ indicates planar, cylindrical, and spherical shaped membranes, respectively. When the membrane is bent, the changes of polymer configuration gives rise to the local extension of the polymer chain. Here the local chain extension at a height r is characterized by $\mathrm{d}r/\mathrm{d}n$, where the variable n denotes the current monomer. This local extension is related to local density profile of monomers $c_p(r)$ as (*2*)

$$c_p(r) = \frac{\mathrm{d}n}{s(r)\mathrm{d}r}. \tag{S2}$$

Then, the thickness of the brush, L_{brush}, is found from the conservation condition (the constraint of conservation of the total number of monomers N) (*2*),

$$\int_{R_t}^{R_t+L_{\text{brush}}} c_p(r)s(r)\mathrm{d}r = N. \tag{S3}$$

As a result, in the brush regime, since the electrostatic effects are excluded, the energy contribution original from the glycocalyx polymers including two terms (*1*, *2*): the elastic energy of the polymer chain ($F_{\text{glycocalyx}}^{\text{elastic}}$) and the free energy caused by the excluded volume interactions of polymer monomers ($F_{\text{glycocalyx}}^{\text{EV}}$). Based on the hypotheses in the main text, according to Ref. (*2*), the elastic energy per chain in the brush can be presented as

$$\begin{aligned} F_{\text{glycocalyx}}^{\text{elastic}} &= \int_{R_t}^{R_t+L_{\text{brush}}} f_{\text{glycocalyx}}^{\text{elastic}} s(r)\ \mathrm{d}r \\ &= \int_{R_t}^{R_t+L_{\text{brush}}} \frac{3k_BT}{2a^2c_p(r)s^2(r)} s(r)\ \mathrm{d}r, \end{aligned} \tag{S4}$$

where a is the monomer length and $f_{\text{glycocalyx}}^{\text{elastic}}$ is the elastic energy density of the polymer chain. Within the mean-field approximation, the energy density of the excluded volume interactions (van der Waals interactions) between monomers can be modeled in terms of the virial expansion

$$f_{\text{glycocalyx}}^{\text{EV}} = k_BT[vc_p^2(r) + wc_p^3(r) + ...] \tag{S5}$$

where v and w are the second and third virial coefficient, respectively. As a result, the excluded volume interactions between monomers per chain in the brush is given by

$$F_{\text{glycocalyx}}^{\text{EV}} = k_BT \int_{R_t}^{R_t+L_{\text{brush}}} [vc_p^2(r) + wc_p^3(r) + ...]s(r)\ \mathrm{d}r. \tag{S6}$$

In subsequent analysis, the cubic and higher terms are neglected. Therefore, the sum of Eq. (S4) and Eq. (S6) yields the energy contribution of glycocalyx polymers

$$\begin{aligned} F_{\text{glycocalyx}} =& N_p\left(F_{\text{glycocalyx}}^{\text{elastic}} + F_{\text{glycocalyx}}^{\text{EV}}\right) \\ =& N_pk_BT \int_{R_t}^{R_t+L_{\text{brush}}} \left[\frac{3}{2a^2c_p(r)s^2(r)} + vc_p^2(r)\right] s(r)\ \mathrm{d}r, \end{aligned} \tag{S7}$$

where N_p is the number of polymer chains grafted on the membrane.

In order to calculate the free energy, we need to further determine the local concentration of monomers $c_p(r)$ and the brush thickness L_{brush}. On a planar membrane surface, the planar brush area per chain, $s(r)$, is constant, i.e., $s(r) = s$. Minimizing the free energy of a planar brush $F_{\text{glycocalyx}}$ with respect to c_p by using $c_p(r)\frac{\delta f[c_p(r),r]}{\delta c_p(r)} - f[c_p(r), r] = 0$ leads to the equilibrium polymer concentration c_p^{flat} (see Ref. (*2*) for detailed steps)

$$c_p^{\text{flat}} = \left(\frac{3}{vs^2a^2}\right)^{\frac{1}{3}}. \tag{S8}$$

Here, $f[c_p(r), r] = f^{\text{elastic}}_{\text{glycocalyx}} + f^{\text{EV}}_{\text{glycocalyx}}$. Using the conservation condition defined by Eq. (S3) yields the brush thickness $L^{\text{flat}}_{\text{brush}}$ (see Ref. (*2*) for detailed steps)

$$L^{\text{flat}}_{\text{brush}} = N\left(\frac{va^2}{3s}\right)^{\frac{1}{3}}, \tag{S9}$$

where we can see that the thickness of polymer brush is proportional to the total number of monomers N. Hereafter, in our model, we use the number of monomers, N, to capture the length of the polymer. Note that the relationship for c_p^{flat} and $L^{\text{flat}}_{\text{brush}}$ are special cases of electrically neutral brushes as described in (*2*). Substituting Eq. (S8) and Eq. (S9) into Eq. (S7) yields the energy contribution of glycocalyx polymers on a planar membrane surface

$$\frac{F^{\text{glycocalyx}}_{\text{flat}}}{\pi\kappa} = N_p\frac{9N}{2\pi\beta\kappa}\left(\frac{v}{3sa}\right)^{\frac{2}{3}}. \tag{S10}$$

Thus, even for a flat membrane, the energy contribution by the glycocalyx is directly proportional to the extent of grafting N_p and the length of the polymer brush N.

On a spherical or a cylindrical membrane surface, the corresponding monomer density profile $c_p(r)$ and brush thickness L_{brush} are, respectively, expressed as (see Ref. (*2*) for a detailed calculation)

$$c_p(r) = \begin{cases} \left[\dfrac{3}{vs^2(r)a^2}\right]^{\frac{1}{3}} = c_p^{\text{flat}}\left(\dfrac{R_s}{r}\right)^{\frac{4}{3}}, & \text{Spherical} \\ \left[\dfrac{3}{vs^2(r)a^2}\right]^{\frac{1}{3}} = c_p^{\text{flat}}\left(\dfrac{R_t}{r}\right)^{\frac{2}{3}}, & \text{Cylindrical} \end{cases} \tag{S11}$$

and

$$L_{\text{brush}} = \begin{cases} R_s\left(1 + \dfrac{5}{3}\cdot\dfrac{L^{\text{flat}}_{\text{brush}}}{R_s}\right)^{\frac{3}{5}} - R_s, & \text{Spherical} \\ R_t\left(1 + \dfrac{4}{3}\cdot\dfrac{L^{\text{flat}}_{\text{brush}}}{R_t}\right)^{\frac{3}{4}} - R_t, & \text{Cylindrical} \end{cases}. \tag{S12}$$

Similarly, substituting Eq. (S11) and Eq. (S12) into Eq. (S7) leads to the energy contribution of glycocalyx polymers on a spherical or a cylindrical membrane surface as

$$\begin{aligned}\frac{F^{\text{cap}}_{\text{glycocalyx}}}{\pi\kappa} =& \frac{N_p^{\text{cap}}(F^{\text{elastic,cap}}_{\text{glycocalyx}} + F^{\text{EV,cap}}_{\text{glycocalyx}})}{\pi\kappa} \\ =& N_p^{\text{cap}}\frac{9R_t}{2\pi\beta\kappa}\left(\frac{3\sqrt{v}}{\xi a^2}\right)^{\frac{2}{3}}\left[\left(1 + \frac{5N}{3R_t}\cdot\left(\frac{va^2}{3\xi^2}\right)^{\frac{1}{3}}\right)^{\frac{1}{5}} - 1\right], \qquad \text{Spherical}\end{aligned} \tag{S13}$$

and

$$
\begin{aligned}
\frac{F_{\text{glycocalyx}}^{\text{tube}}}{\pi\kappa} =& \frac{N_p^{\text{tube}}(F_{\text{glycocalyx}}^{\text{elastic,tube}} + F_{\text{glycocalyx}}^{\text{EV,tube}})}{\pi\kappa} \\
=& N_p^{\text{tube}} \frac{9R_t}{4\pi\beta\kappa} \left(\frac{3\sqrt{v}}{\xi a^2}\right)^{\frac{2}{3}} \left[\left(1 + \frac{4N}{3R_t} \cdot \left(\frac{va^2}{3\xi^2}\right)^{\frac{1}{3}}\right)^{\frac{1}{2}} - 1\right], \qquad \text{Cylindrical} \qquad \text{(S14)}
\end{aligned}
$$

where N_p^{cap} and N_p^{tube} are the number of polymer chains that grafted on the spherical cap and the cylindrical tube. Combining Eqs. (S13) and (S14), and using the relation $\chi = L_t/R_t$ defined in the main text reduces to Eq. (7) in the main text.

2 Contributions from the different energy components

To reveal the mechanisms of tube formation regulated by glycocalyx, different energy components contribute to the total free energy profiles in Fig. 2 are plotted as a function of shape parameter χ, as shown in Fig. S2. Figure. S2 demonstrates that membrane bending is governed by a balance among these energy players.

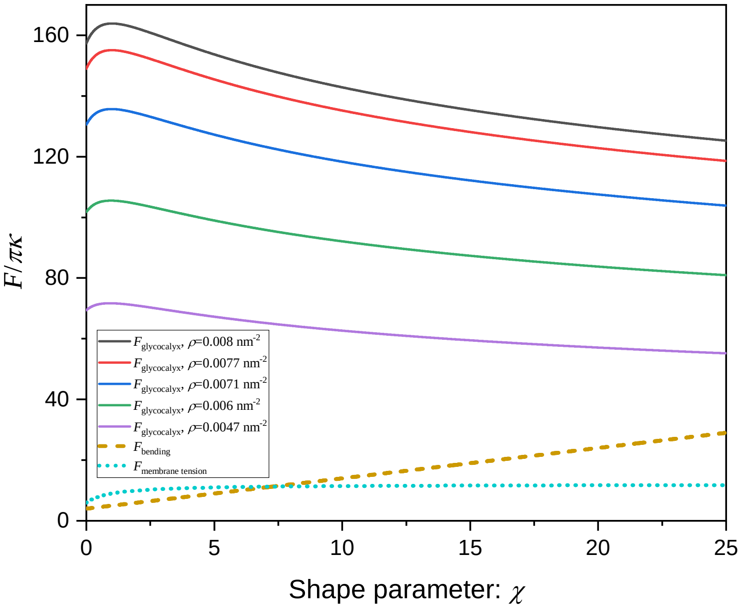

Figure S2: Different types of energy profiles including energy contribution associated with glycocalyx $F_{\text{glycocalyx}}$, bending energy F_{bending}, and tension energy $F_{\text{membrane tension}}$ as a function of the shape parameter χ for different grafting densities.

3 Phase diagrams on the (κ–σ), (λ–c_0), and (f–c_0) planes

To gain more insight into the effects of the membrane properties and spontaneous curvature, line tension, and actin force on tube formation, three phase diagrams on the κ–σ, λ–c_0, and f–c_0 plane are constructed, as shown in Fig. S3. Figure S3(a) shows that forming a tube is more favorable under lower membrane bending rigidity and membrane tension. Figure S3(b) and (c) confirmed that longer and thinner tube can be formed with the assistance of line tension, spontaneous curvature, and actin force.

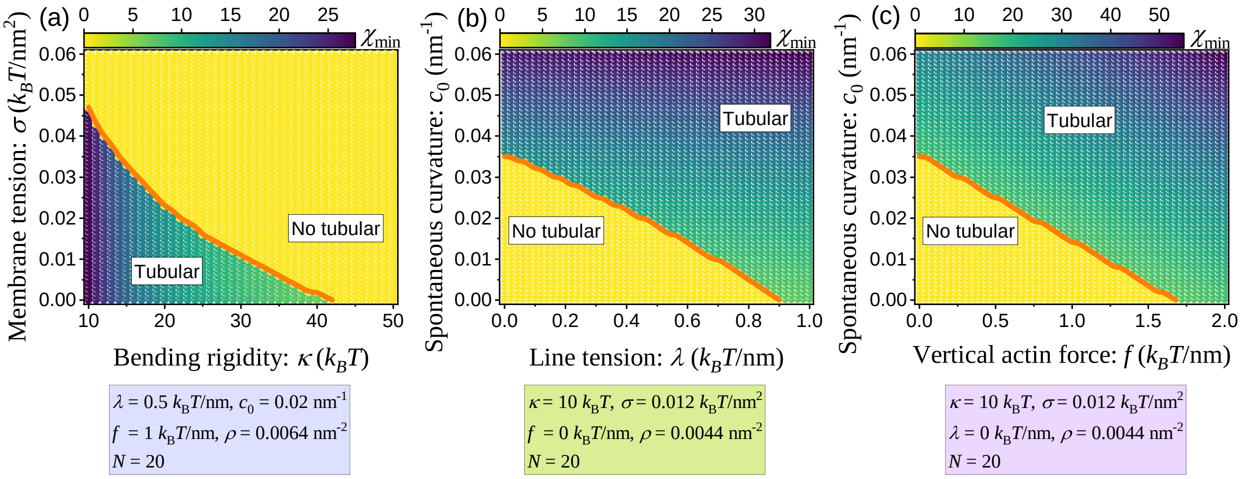

Figure S3: (a) Heatmap of optimal shape parameter $\chi_{\min}$ as a function of membrane bending rigidity κ and membrane tension σ. (b) Contour plot of optimal shape parameter $\chi_{\min}$ as a function of line tension λ and spontaneous curvature c_0, where the color bar represents the magnitude of the optimal shape parameter. (c) A two-dimensional phase diagram on the (f–c_0) plane characterizes the interrelated effects of vertical actin force and spontaneous curvature on the membrane shape.

References

1. C. R. Shurer *et al.*, *Cell* **177**, 1757–1770.e21, DOI `https://doi.org/10.1016/j.cell.2019.04.017` (2019).

2. E. B. Zhulina, T. M. Birshtein, O. V. Borisov, *Eur. Phys. J. E* **20**, 243–256, DOI `10.1140/epje/i2006-10013-5` (2006).
